# Advancing human sensorimotor research through modular robotics: the case of the HRX-1

**DOI:** 10.1098/rstb.2024.0464

**Published:** 2026-09-17

**Authors:** Ildar Farkhatdinov, Joshua Brown, Aaron Yurkewich, Etienne Burdet

**Affiliations:** ^1^ King's College London, London, UK; ^2^ Human Robotix Ltd, UK; ^3^ Imperial College of Science Technology and Medicine, London, UK; ^4^ Ontario Tech University, Oshawa, Canada

**Keywords:** rehabilitation robotics, assistive technology, sensorimotor control

## Abstract

The HRX-1 is a single-degree-of-freedom, direct-drive, modular and reconfigurable robotic device designed for investigating human sensorimotor control. By examining its use, this case study highlights the opportunities offered by modular, portable and user-centred open-source design approaches for sensorimotor diagnostics and basic neuromechanics research. The robot's architecture enables simple and rapid reconfiguration, allowing researchers to explore a wide range of experimental questions. When combined with muscle electromyography, functional electrical stimulation and electroencephalography, the system enables investigation of how muscles respond to external forces and how the brain coordinates the responses. These multimodal approaches also support the development of novel movement-assistive strategies for neuromotor rehabilitation. Clinical applications include studies of brain activity patterns in children with dystonia, upper-limb rehabilitation after stroke and therapy for patients with functional neurological disorders.

This article is part of the discussion meeting issue ‘Digital healthcare for the management of functional neurological disorders’.

## 1. Introduction

Robotic and haptic interfaces have become valuable tools for investigating the human sensorimotor control system because they enable precise, repeatable manipulation of mechanical stimuli and accurate measurement of motor responses. These systems allow researchers to systematically investigate sensorimotor control and learning under controlled conditions that are difficult to control well in natural movement studies. By providing programmable force and position feedback, robotic devices also help quantify how the nervous system integrates sensory information for motor planning and correction, advancing computational models of human sensorimotor control [1–8]. In clinical contexts, such interfaces can support neuromechanics diagnostics and have the potential to transform neurorehabilitation through adaptive, assist-as-needed therapy and objective assessment of motor recovery after stroke or spinal cord injury (SCI) [9]. Furthermore, haptic feedback technologies can be used to enhance patient engagement and promote neuroplasticity through interactive, task-specific training. Overall, robotics and haptics bridge neuroscience and clinical rehabilitation, supporting neuromechanics understanding of sensorimotor functions while offering evidence-based tools to assist recovery of movement and function.

Robotic devices become common in clinical research on neurological conditions, such as stroke, SCI and functional neurological disorders (FND), providing objective and quantifiable measures of motor function and sensorimotor integration [10]. They enable precise control of movement kinematics, forces and timing, allowing standardized assessment of motor deficits and recovery trajectories that are difficult to capture with conventional clinical scales [11]. In stroke rehabilitation, robotic platforms have been used to characterize impairments in reaching, grasping and gait, while also monitoring changes in motor performance over time, thereby facilitating evaluation of intervention efficacy [12,13]. In SCI, exoskeletons and gait trainers provide repeatable locomotor patterns that can be coupled with physiological measurements, such as electromyography (EMG) or neuroimaging, to study neuroplasticity and sensorimotor reorganization [14,15]. For FND, robotics can provide required movement assistance to restore a patient's mobility [16]. Robotic systems support adaptive and intensive training protocols that can be systematically varied, facilitating the study of motor learning and recovery mechanisms [17]. Overall, their clinical integration provides high-resolution, reproducible neuromechanics data to inform targeted rehabilitation strategies [18,19]. Various manipulanda-type robots for investigating human sensorimotor control have been developed. Examples for upper limb research include large grounded multi-degree-of-freedom robotic exoskeletons [20,21], end-effector manipulanda robots [22,23] and desktop-scale robots [24,25]. However, their broader adoption in research and clinical environments has been limited by a lack of portability, and insufficient modularity preventing multifunctionality. Most of these systems rely on multiple motors and large structures to accommodate different joints; resulting in heavy, costly setups that require substantial space and constrain user posture. This limits accessibility to researchers, clinicians and patients with comorbidities [26].

This HRX-1 robotic system-based case study highlights the importance of modular, portable and open-source design approaches in robotics for studying human sensorimotor control and developing neurorehabilitation technologies. The following sections present the HRX-1 robot and illustrate its use in both fundamental and clinical neuroscience research enabled by its modularity, reconfigurability and portability.

## 2. Modularity, portability and open-source design in robotics for neuro-rehabilitation and assistive technologies

A modular design approach in rehabilitation and assistive robotics enables flexible, scalable and customizable systems that can be tailored to the diverse needs of patients and clinical applications. By designing robots as a collection of interchangeable modules—such as actuation units, sensors and control components—devices can be adapted to different limbs, impairments or therapeutic goals without complete redesigns [27,28]. This flexibility can accelerate research and clinical translation by allowing rapid prototyping and iterative testing of new control algorithms and interfaces. From a clinical perspective, modularity facilitates device personalization, allowing the level and type of assistance to evolve with patient recovery, thereby supporting adaptive therapy paradigms. It also enables lighter, more ergonomic devices with adjustable mechanical and control modules that improve comfort, compliance and engagement during long-term use [29]. In addition, shared components and standardized interfaces reduce maintenance costs and simplify upgrades. Modular architectures further enhance interoperability between robots, sensors and stimulation technologies, paving the way for hybrid systems that combine, for example, robotic assistance with functional electrical stimulation or biofeedback [30]. Overall, modularity promotes both technological innovation and clinical effectiveness through adaptable and patient-centred solutions.

The concept of modular robotics in rehabilitative and assistive motion systems has been explored across various distinct hardware configurations. For example, Kumar *et al.* present the dual-arm RECUPERA Exoskeleton with a high-level of modularity and decentralized control, supporting wheelchair-mounted or full-body configurations for upper-body stroke rehabilitation [31]. Jayaraman *et al.* demonstrate a modular hip exoskeleton that improves walking function and reduces sedentary time in older adults, illustrating how modularity supports adaptability to gait-related impairments [32]. For upper-limb applications, the ‘modular synthesis’ approach by Nehrujee *et al.* generates multiple device configurations supporting motion in different degrees-of-freedom (DoF) [24]. Other works focus more on modular design and hardware architecture, providing mechatronic design and analysis of modular shoulder–elbow or hand–wrist robots for rehabilitation [24,33], while Nesler *et al.* introduce the modular back-drivable lower-limb unloading exoskeleton (M-BLUE) with hip/knee modular assist configurations [34]. While these systems demonstrate adaptability, personalization and reconfigurability, many remain at the prototype of early validation stage rather than large-scale clinical deployment.

Portability and compact design are other critical factors in the development of robotic devices for neuro-rehabilitation and assistive technology [35]. Portability directly influences usability and accessibility across clinical, research and home environments. Devices that are easy to transport and install without specialized infrastructure are more likely to be adopted in clinical practice. Lightweight and compact systems reduce logistical and spatial constraints, enabling deployment across multiple sites and facilitating studies in diverse settings. These characteristics are particularly important for resource-limited environments, where portability can significantly broaden access to rehabilitation technologies.

Open-source robotics for neuro-rehabilitation and assistive devices has grown significantly in recent years, enabling low-cost, customizable and reproducible solutions for clinical and research use. While initially more common in software, open-source approaches are increasingly applied to hardware design. Examples include wearable neuroprosthetic components [36], open-source hand rehabilitation exoskeletons [37], the modular OpenExo platform with open computer-aided designs (CAD) [38] and soft fabric-based hand exoskeleton [39]. Open-source design promotes accessibility and reproducibility, fostering community-driven development of scalable and adaptable systems, and supporting collaboration across research and clinical communities [36,40].

## 3. Overview of the HRX-1 robot and its modularity

**Figure 1.**
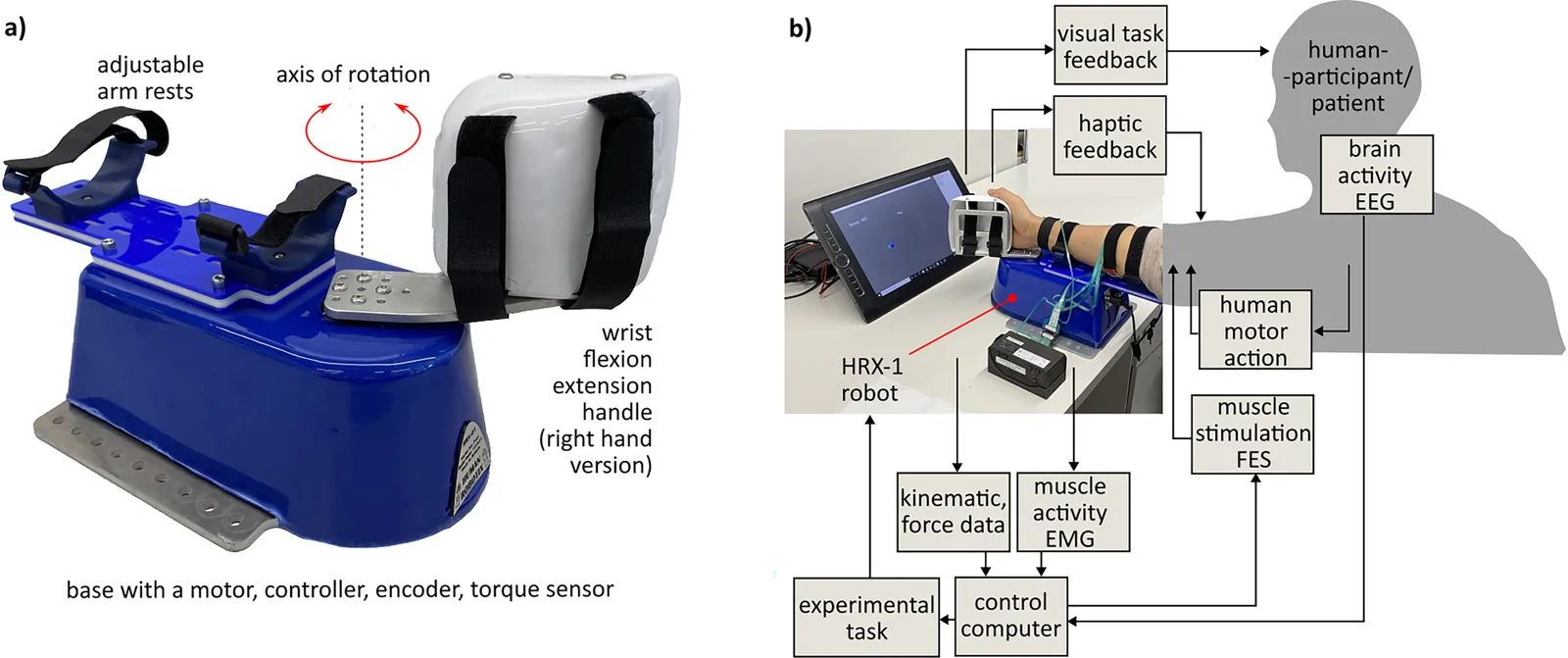
(a) Wrist configuration of the HRX-1 robot. (b) Schematic block diagram illustrating the use of HRX-1 wrist robot in movement training task with visuo-haptic feedback, functional electric stimulation (FES), muscle electromyography (EMG) and electroencephalography (EEG).

Concepts of modularity, portability and open-source design guided the development and use of the HRX-1 robot across all applications described in this article. HRX-1 is a modular, portable and reconfigurable partially open-source robotic system for investigating human sensorimotor control and prototyping neuro-rehabilitation and assistive technologies, as well as general-purpose physical human–robot interaction and control strategies. Figure 1 shows the HRX-1 configured as a desktop wrist exoskeleton. The HRX-1 robot was developed by Human Robotix Ltd^1^ and inspired by the *Hi5* dual robotic system dedicated to robot-mediated human–human and bimanual interaction investigation [41]. However, the *Hi5* robot is not portable and not suitable for experiments in clinical environments owing to its room-scale size, large weight and mechatronic complexity.

To address these limitations, HRX-1 was designed with portability and modularity as core requirements. Its compact and lightweight design facilitates storage, transport and installation. Modularity is achieved through the common hardware interfaces (modular attachments) and software components (control interfaces). The control software is made open source to enable development of new experimental applications and integration with other systems. Hardware documentation for modular elements is also open source to support the development of new human–robot interfaces.

HRX-1 implements programmable single-degree-of-freedom rotation using a low-friction brushless direct-drive motor (600W Maxon EC90) with integrated angular position and torque sensors. The actuator, sensors and control electronics are mounted on a custom lightweight metal frame enclosed in a plastic casing (figure 1a). This direct-drive actuation offers relatively high torque generation, as well as accurate position and velocity measurements across multiple control modes. The nominal torque is 1.6 Nm, with a peak of 4 Nm (up to 15 s), and a motion range of ±140^∘^. The device fits within 28 × 20 × 11.5 cm and weighs less than 4 kg. The default inertia (motor with coupler) is 5100 gcm^2^, although it depends on the handle configuration. The motors’ mechanical time constant is 12 ms.

Motion is controlled via a Maxon EPOS4 controller, providing closed-loop position, velocity and current control. The internal current control loop runs at 25 kHz, and the electrical time constant is approximately 1 ms. Current control is particularly relevant for rehabilitation, neuromechanical identification, and physical human–robot interaction applications. The system is powered by a standard 19.5 V adapter (15 A limit) and incorporates multiple safety features, including adjustable mechanical end-stops, an emergency stop button and software limits on torque and velocity. Electronic and power components are shielded where necessary to reduce electromagnetic interference, which is critical for use with EMG and EEG systems.

HRX-1 supports two software programming/execution modes: (i) Matlab-based scripting (non-real time, approx. 100–150 Hz, USB communication between control PC and robot) and (ii) real time using an embedded controller with CAN communication (1 kHz control loop). The real-time mode enables high-quality haptic rendering and precise motion control. Control bandwidth depends on configuration; for example wrist force control bandwidth is approximately 150 Hz, and position control bandwidth is approximately 12 Hz. This allows rendering of a wide range of force fields, from rapid perturbations to fine haptic textures. In summary, users can select between ease-of-use (Matlab API) and high-performance real-time control (C/C++ API with CAN interface). More technical details about the HRX-1 can be found in Brown *et al.* [42].

Table 1 compares key performance characteristics of HRX-1 with representative commercial, academic and open-source devices from 1993 to 2024, highlighting its unique combination of portability, versatility and relatively high torque output. In its default (wirst) configuration, HRX-1 offers more torque than comparable 1-DoF wrist-based robots and significantly lower physical size and weight, while offering the option to add the capability to work with other upper and lower limb joints via a system of reconfigurable modules. It is however not able to compete with the levels of torque available from larger elbow and shoulder oriented devices without external gearing, as these systems are designed to resist larger forces and torques from the user/wearer.

**Table 1.** Comparison of key performance characteristics of the HRX-1 robot with representative commercial, academic and open-source systems. –, indicates that a characteristic is not reported in the literature or product specifications.

**device**	**year**	**#****DoF**	**maximum torque (Nm)**	**joint**	**size (cm)**	**mass (kg)**	**position resolution**
MIT-MANUS [43]	1993	5	1.43	wrist and arm	–	–	–
KinArm [44]	1999	2	16.5	shoulder and elbow	100 × 100	–	0.0006^∘^
ABLE [20]	2008	4	18 (shoulder), 13 (elbow)	shoulder and elbow	–	13	–
Wristbot [45]	2009	3	1.53	wrist	–	–	–
vBot [46]	2009	3	16, 3 (wrist)	wrist, planar arm	approx. 80 × 100 × 150	–	–
Hi5 [41]	2011	2	15	bimanual, wrist	120 × 90 × 80	20	0.072^∘^
HapKit [47]	2014	1	0.25	–	20 × 15 × 20	–	–
3DOM [21]	2014	3	32.8 (shoulder), 3.68 (wrist)	shoulder, elbow, wrist	110 × 86 × 126	–	0.03 mm
H-Man [48]	2014	2	–	shoulder and elbow	40 × 22 × 15	–	–
ArmAssist [22,49]	2017	3	–	shoulder and elbow	–	10	–
EMU [50]	2017	3	48.5	upper limb	–	–	–
HRX-1 [42]	2021	1	4	wrist, elbow, ankle	28 × 20 × 11.5	4	0.014^∘^
PLUTO [24]	2021	1	3.38	wrist or hand	–	–	–
TSA wrist robot [25]	2022	1	2.4	wrist	–	–	–
EDUSA PRO-R [23]	2024	3	36.24–108.15	wrist	75 × 129 × 90	65	0.00006^∘^

Despite the benefits of direct-drive actuation, torque capacity is limited compared to larger systems (continuous approx. 1.6 Nm). In addition, the single-degree-of-freedom design constrains studies to single-joint movements. Although extensions to elbow and ankle joints are possible, multi-joint coordination requires more complex systems. Multi-DoF setups using multiple HRX-1 units are feasible but remain limited in overall torque. Finally, as a grounded desktop device, HRX-1 is not suitable for wearable applications.

**Figure 2.**
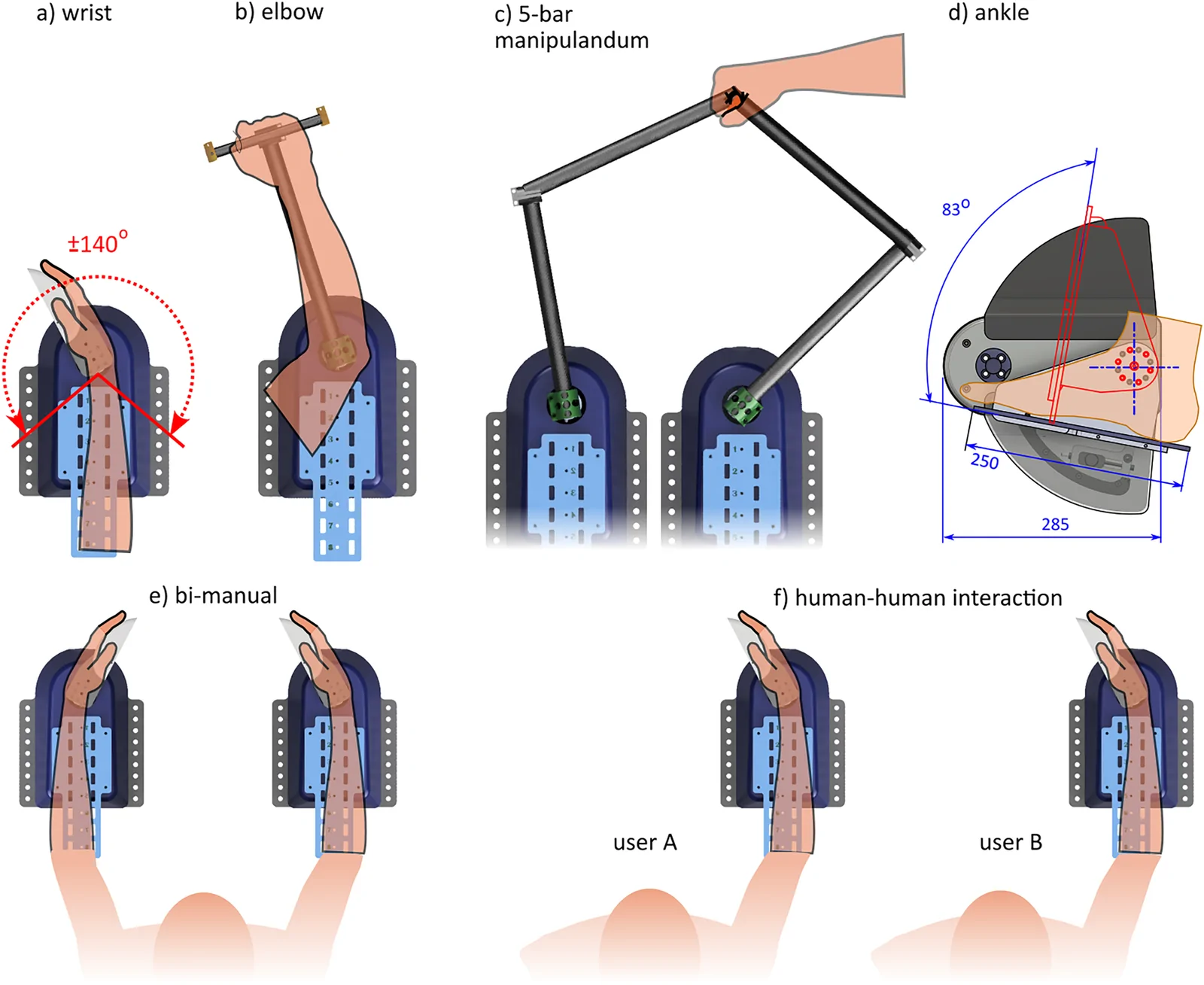
Examples of modular configurations of the HRX-1 robot: (a) wrist flexion/extension; (b) elbow flexion/extension; (c) five-bar end-effector manipulandum; (d) ankle plantar-/dorsi-flexion; (e) bimanual wrist configuration; (f) configuration for multi-human interaction research.

Figure 2 illustrates possible configuration options enabled by the HRX-1: wrist (a), elbow (b), manipulandum (c), ankle (d), bimanual (e) and human–human interaction (f). Modularity is achieved through: (i) interchangeable handles/end-effectors attached to the motor's shaft; (ii) combination of multiple HRX-1 units to form different mechanisms; and (iii) flexible mounting on various bases and orientations. Application-specific attachments include wrist/elbow/foot interfaces, arm supports, torque-multiplying gearboxes, and other mechanisms. The wrist handle consists of a lightweight aluminium frame, an ergonomic open palm support (minimizing forearm activation) and Velcro straps for secure fixation [21].

Typical configurations of the HRX-1 robot include:

— *Wrist* flexion/extension using the default arm support and open-palm handle, widely used in neuromechanics studies [51,52].
— *Elbow* flexion/extension via an additional linkage and arm/shoulder support, enabling studies based on established elbow neuromechanics literature [53–55].
— *Manipulandum*: a planar two-DoF five-bar mechanism using two HRX-1 units, enabling investigation of nonlinear arm dynamics and redundancy [2,3,6,56]. The adjustable distance between the HRX-1 robots and the lengths of the mechanism's links define the workspace and manipulability of the robot [21,57].
— *Bimanual* interface: two synchronized units for studying coordinated bimanual interaction (e.g. [58]).
— *Human–Human* physical interaction: two or more units connected via software to study dynamic interaction between participants (e.g [59,60]).
— *Ankle*: dorsiflexion/plantarflexion using a floor-mounted setup with torque amplification via a cable-driven gearbox [61].
— *Tactile stimulation*: integration of additional haptic devices (e.g. soft or vibrating interfaces) to study combined tactile and kinaesthetic feedback [62,63].

Systems configuration can be modified with minimal effort. Single-robot upper-limb setups can be adjusted within 1–2 minutes using standard hex keys. More complex configurations (e.g. ankle or multi-robot systems) require additional components and synchronization but can typically be completed within minutes. Multi-robot setups may use a shared CAN bus for real-time control (1 ms synchronization). Software updates and recalibration are required to account for configuration-specific dynamics, such as changes in inertia and range of motion.

## 4. Application cases

### (a) Human motor control: muscle activation, robotic assistance and functional electrical stimulation

The HRX-1 robotic platform facilitates the efficient implementation of complex and adaptive experimental paradigms designed to investigate human sensorimotor control. The system can be readily integrated with EMG and functional electrical stimulation (FES) technologies, enabling comprehensive experimental studies that examine movement neuromechanics at joint, muscle and—when combined with, e.g. EEG—brain levels, as shown schematically in figure 1b.

Integration of surface EMG with the HRX-1 robot is described in Brown *et al.* [42]. The wrist configuration of the robot was used in combination with 32-channel high-density surface EMG to measure forearm muscle activation (wrist flexor and extensor muscle groups). Fifteen healthy participants performed tasks seated in which they maintained or resisted wrist flexion and extension torques applied by the robot. The EMG data analysis showed greater activation of flexor muscles when resisting flexion torque and extensor muscle groups dominated during extension. The results suggest that spatial recruitment patterns of wrist-forearm muscles remain stable across torque levels. The study demonstrated that HRX-1 provides a reliable platform for controlled measurement of muscle activation under externally applied wrist torques.

In a neuroscience study [64], HRX-1 was used to explore how humans use muscle co-contraction to cope with uncertainty during movement. Computational modelling predicted that as the probability of external disturbances increases, optimal muscle co-contraction rises approximately logarithmically. To test this, a wrist-pointing experiment was conducted in which the robot applied unpredictable torque perturbations probabilities (of 0%, 25%, 50%, 75%, 100%). EMG measurements showed that co-contraction increased significantly under intermediate uncertainty (25%–75%) compared to both deterministic conditions (0%, 100%). These findings support the interpretation of co-contraction as an optimal feedforward impedance control strategy.

In addition to wrist configurations, HRX-1 was used in an ankle setup with a modular transmission to explore whether it can generate resistive/assistive torques at the ankle joint [65]. A cable-driven, low-friction, low-inertia transmission was added to increase torque output. The designed ankle interface was backdrivable, capable of producing up to 40 Nm, and included gravity compensation to enable symmetric plantar/dorsiflexion in free movement. The robot was placed on the floor in front of a seated user, whose right foot was attached to a custom ankle-foot platform. Haptic virtual floor feedback was tested while users resisted imposed ankle rotations. Surface EMG from the Gastrocnemius Medialis and Tibialis Anterior showed a clear linear relationship between applied torque and muscle activation, demonstrating physiologically consistent responses.

HRX-1 can be used as a research tool to explore and test motion assistance for neuromotor rehabilitation. For example, Widanage *et al.* [66] introduced an optimization-based controller incorporating vibration constraints to reduce pathological tremor, alongside position and velocity constraints. Results showed improved performance through tremor attenuation while maintaining positional accuracy. This work highlights how richer constraint sets can yield safer, more patient-specific assistance.

The study of Hafs *et al.* [67] presents a motion assistive controller based on a game-theoretic framework. The human and robot were modelled as agents in a finite-horizon differential game minimizing tracking error and control effort. The control was computed through an optimization process allowing the robot to adapt assistance in real time to the user's behaviour and goals. Experiments conducted with the HRX-1 robot in wrist configuration showed improved trajectory tracking, reduced user effort, and smoother interaction compared to standard approaches not considering the user, with rapid convergence of estimated human cost parameters.

Multiple studies have explored hybrid assistance combining HRX-1 with FES. In the system of Kavianirad *et al.* [68], an adaptive filter estimates voluntary torque from EMG contaminated by FES artefacts, enabling closed-loop assistance based on user effort. Results demonstrated reliable torque estimation in healthy participants.

In Cazenave *et al.* [69], the effect of robotic assistance, FES and their combination were evaluated in a tracking task. Performance improved with robotic assistance alone or combined with FES, while FES alone showed no benefit. EMG analysis indicated reduced muscle activation under robotic assistance, with similar fatigue levels across conditions. These findings suggest hybrid robot + FES systems may reduce physical and cognitive strain during rehabilitation. This paradigm was extended in Cazenave *et al.* [70], which examined fatigue under voluntary, FES-only and combined conditions. A recent study [71] combined HRX-1 with high-density EMG and EEG to investigate cortico-spinal propagation of beta-band activity. Results showed temporal alignment between cortical beta bursts (13–30 Hz) and motor-unit activity across multiple muscles, suggesting a robust, large-scale propagation mechanism.

### (b) Clinical research

This section presents clinical case studies in which the HRX-1 was used to generate controlled upper limb movements and collect corresponding data.

A study on proprioceptive modulation of cortical connectivity in young people with dystonia is presented in Sakellariou *et al.* [72]. HRX-1-generated passive wrist extension combined with EEG revealed exaggerated theta-band connectivity in dystonia, supporting its characterization as a network disorder with abnormal sensorimotor integration. Follow-up work [73] showed reduced *α* / *μ* desynchronization and synchronization responses in dystonia patients, indicating impaired cortical processing of proprioceptive input.

A pilot study on sub-acute stroke patients [74] evaluated a hybrid HRX-1 + FES rehabilitation system. Most participants showed improved tracking and smoother motion under assistance. Benefits appeared greater for moderate-to-severe impairment, and users reported good comfort and engagement, supporting feasibility of early-stage intervention.

A study on FND [75] introduced an extended reality (XR)-based biofeedback platform integrating HRX-1. A Delphi survey and co-design workshop identified key requirements including customizability, real-time feedback, comfort and accessibility. Feedback emphasized ergonomic hardware, immersive content and adjustable assistance, alongside cost and home-use considerations.

### (c) Haptic rendering and tactile perception

An earlier HRX-1 prototype was used to develop haptic interfaces for tactile perception research. In Wilhelm *et al.* [63], a fingerpad device with interchangeable textures was mounted on the robot, which controlled precise movement of stimuli across the fingertip. The system enabled controlled delivery of normal forces and lateral motion, allowing systematic measurement of detection thresholds and spatial discrimination. Results showed variations in sensitivity across hand regions consistent with mechanoreceptor distributions.

In Brown & Farkhatdinov [62], the wrist handle was replaced with a soft haptic joystick capable of vibration, shape change and stiffness modulation. The joystick was synchronized with HRX-1 torque feedback to render complex haptic environments. Evaluation via vibrotactile signal analysis demonstrated the potential of combining soft haptics with force-feedback robotics for rich, dynamic haptic rendering.

## 5. Conclusion

This overview of HRX-1 applications in both basic and clinical neuromechanics research demonstrates that the robot's primary role is to generate controlled force fields and proprioceptive inputs for the upper limbs of human participants. When integrated with EMG and EEG systems, HRX-1 enables simultaneous recording of responses at the joint, muscle and cortical levels, a capability that remains difficult to achieve with most experimental and clinical platforms. HRX-1 has been employed in multiple studies investigating how the human brain and neuromuscular system coordinate movement, as well as how robotic assistance can support the development of novel neurorehabilitation approaches.

The modular and portable design of the HRX-1 device, and its reconfigurable architecture, has proven essential for supporting a broad range of research applications. The wrist-module configuration is the most widely used, owing to its ease of deployment in clinical environments, adaptability to user-specific requirements, and ability to provide high-quality experimental data, while enabling comparison with the extensive literature on wrist neuromechanics. The robot's low inertia, minimal friction and gearless direct-drive actuation are critical for ensuring safe, accurate and haptically transparent force and motion generation.

Portability is a central design feature of HRX-1. The robot is compact and lightweight, allowing convenient transport and deployment without specialized equipment. It can be readily assembled and installed in a variety of settings, including research laboratories, clinical environments and participants’ homes. This portability has been instrumental in enabling multiple studies, particularly in space-constrained clinical settings and in research involving stroke, functional neurological disorders and dystonia.

The authors’ engineering and collaborative research experiences indicate that a modular design approach, combined with open-source hardware co-development among engineers, clinicians and neuroscientists, is essential for the successful integration of robotic systems into human sensorimotor research. For neurorehabilitation applications, effective robotic platforms must be portable, reconfigurable and technologically robust—characteristics best achieved through lightweight modular construction, a reduced number of moving parts and ergonomic human–robot interfaces.

Future developments include the introduction of a more powerful actuator to achieve higher torque output while maintaining direct-drive operation. At present, the robot provides approximately one third of the continuous torque generation capacity of healthy adult wrist flexion. Although this is sufficient (and safer!) for most clinical applications, it limits the robot use in tasks requiring high perturbations. Additional developments may include improved multi-unit kinematic configurations enabling more complex and non-planar movements. Finally, while HRX-1 has primarily been used as a research platform for developing novel rehabilitation and sensorimotor assessment methods, further studies are required to evaluate clinical efficacy.

## Data Availability

This article has no additional data.

## Footnote

^1^

http://www.humanrobotix.co.uk

## Theme

One contribution of 12 to a discussion meeting issue ‘Digital healthcare for the management of functional neurological disorders’.
